# Positional assembly of hemin and gold nanoparticles in graphene–mesoporous silica nanohybrids for tandem catalysis†
†Electronic supplementary information (ESI) available. See DOI: 10.1039/c4sc02714k



**DOI:** 10.1039/c4sc02714k

**Published:** 2014-11-24

**Authors:** Youhui Lin, Li Wu, Yanyan Huang, Jinsong Ren, Xiaogang Qu

**Affiliations:** a State Key Laboratory of Rare Earth Resource Utilization and Laboratory of Chemical Biology , Changchun Institute of Applied Chemistry , Chinese Academy of Sciences , Changchun , Jilin 130022 , P. R. China . Email: jren@ciac.ac.cn ; Email: xqu@ciac.ac.cn ; Fax: +86 431 85262656; b Graduate School of the Chinese Academy of Sciences , Beijing , 100039 , P. R. China; c Research Institute for Soft Matter and Biomimetics , Department of Physics & College of Materials , Xiamen University , Xiamen 361005 , P. R. China

## Abstract

Here, for the first time, a hybrid catalyst in which hemin and gold nanoparticles are positioned in spatially separate domains within a graphene–mesoporous silica support is presented. Specifically, monomeric hemin can be anchored on the inner exposed graphene surface of a graphene–mesoporous silica hybrid (GS) *via* π–π stacking interactions. After the assembly, such nanocomposites can function as a peroxidase mimic. Next, gold nanoparticles, which act as artificial glucose oxidase, can be conjugated to the functional NH_2_ groups present on the outer coated silica surface. As a result, the integrated catalysts containing multiple catalytic sites can be used to catalyze sequential reactions, without the aid of true enzymes. This work is an important step forward in positional assembly of biomimetic catalysts for artificially mimicking natural organelles or important chemical transformations in the future.

## Abstract 2

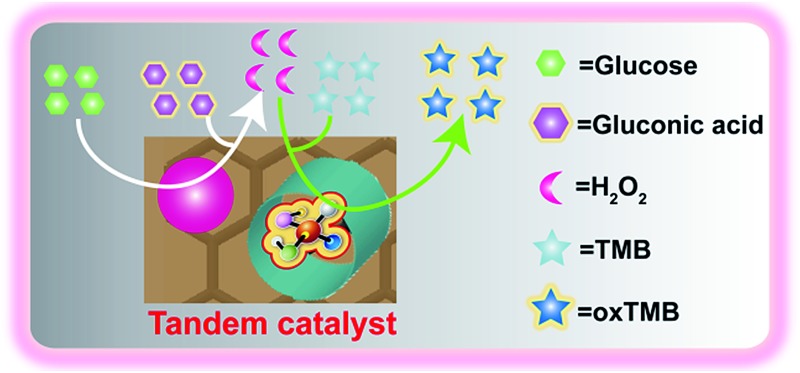
A hybrid catalyst in which two different types of enzyme mimics are positioned in spatially separate domains within a graphene–mesoporous silica support is presented.

## Introduction

In a natural environment, enzymes are almost always spatially confined in crowded and tightly controlled cellular compartments, which can isolate the catalytic cycle, prevent interference and make biomolecular catalysts more efficient.^1^ In order to mimic the natural compartmentalization process, researchers have long directed their attention to enzyme encapsulation or assembly.^2^ Until now, much effort has been focused on using phospholipid liposomes or polymersomes as synthetic nano- or micro-capsules.^3^ Furthermore, to be efficient, the biomolecular catalysts need not only to be presented in a confined reaction space but also positioned at specific sites within subcellular organelles.^3^ To this end, van Hest and collaborators constructed a variety of biohybrid polymersome nanoreactors in which two or more different enzymes were spatially positioned and precisely ordered.^1a,4^ Very recently, through loading different enzyme-containing organelle mimics inside larger polymersomes, they have even successfully created a structural and functional eukaryotic cell mimic.^5^ Recently, Lu *et al.* demonstrated a promising approach by assembling and encapsulating enzymes within a thin polymer shell to form biomimetic enzyme nanocomplexes with precise compositional and spatial controls.^6^

On the other hand, using synthetic systems to simulate the function of natural enzymes has attracted increasing attention for the last few decades.^7^ Among the countless examples arising from these efforts, catalytically active nanomaterials as a new generation of artificial enzymes are particularly impressive and lead to new opportunities in biomedical diagnosis, environmental monitoring, and therapeutics.^8^ Until now, researchers have discovered a number of nano-sized materials that possess unique enzyme-mimicking activities, such as CeO_2_,^9^ Fe_3_O_4_,^10^ gold nanoparticles (AuNPs),^11^ V_2_O_5_,^12^ PtPd–Fe_3_O_4_,^13^ graphene oxide^14^ and graphene nanocomposites.^15^ Nevertheless, creating such “static” artificial enzymes is not sufficient to mimic smart enzymatic systems, just like simply combining individual biomolecules (*e.g.* protein, nucleic acid and lipid) together is not enough to construct a functional cell.^16^ Recently, through the integration of artificial enzymes with natural enzymes, catalytic ensembles with synergic and complementary functions have been achieved.^15b,17^ Such studies take one important step towards mimicking complex natural systems. To mimic nature more completely, it would be desirable not only to explore “static” artificial enzymes, create catalytic ensembles or design functional enzyme complexes with a high level of control over positional assembly, but also to position different types of artificial enzymes (or prosthetic groups) in separate domains.

**Fig. 1:**
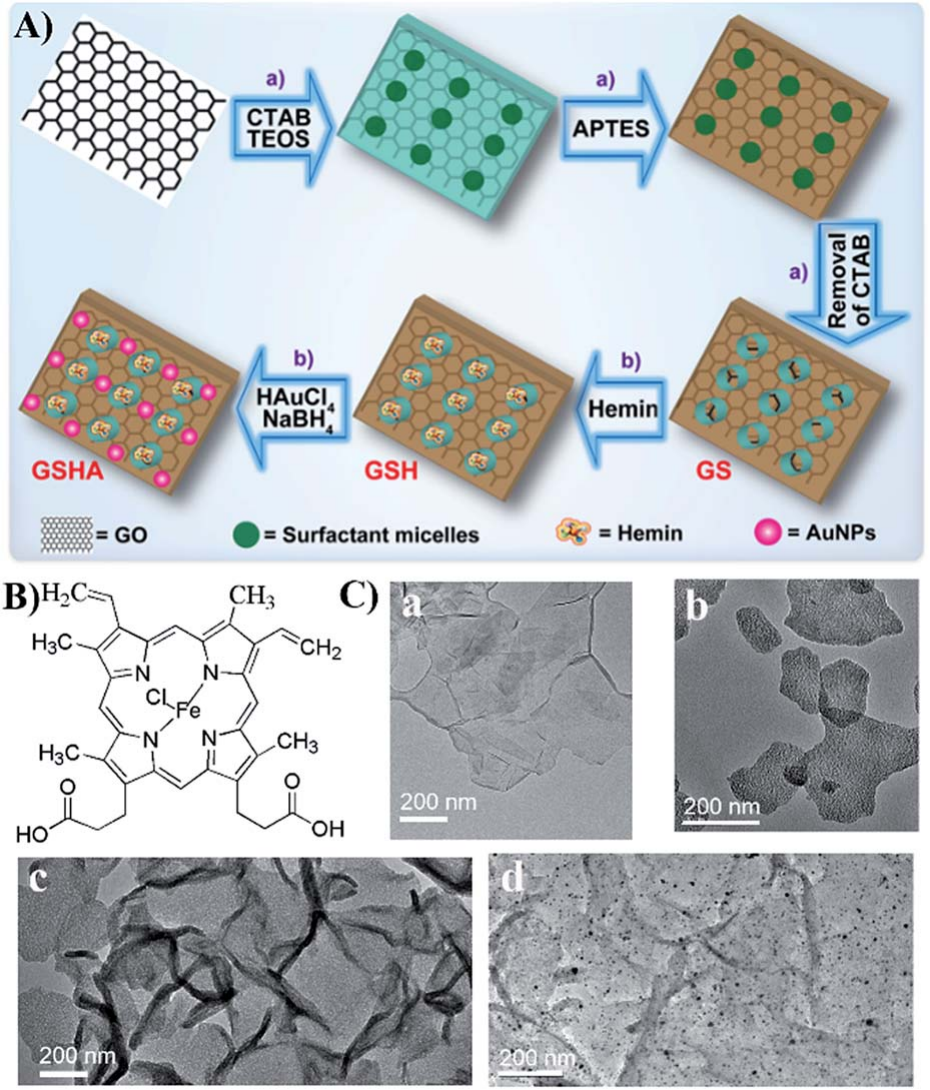
(A) Synthetic strategy for the construction of the GSHA catalyst. (a) Fabrication process for the GS sheets, whose surface is functionalized with amine groups before removing CTAB. (b) Positional assembly of hemin and AuNPs in the GS support. (B) The molecular structure of hemin. (C) TEM images of (a) GO, (b) GS, (c) GSH and (d) GSHA.

Herein, we describe the rational design of robust artificial enzyme nanocomplexes to achieve this aim, as shown in Fig. 1. Specifically, a graphene–mesoporous silica hybrid (GS) was used as a nanocontainer to anchor two artificial enzymes (*i.e.* AuNPs as a glucose oxidase (GO*x*) mimic and hemin as a prosthetic group to mimic peroxidase) at different locations, namely, on the outer surface of coated silica and on the inner surface of exposed graphene, respectively. This environment allows the simple design of an artificial enzymatic reaction system in which AuNPs and hemin can work in tandem catalysis. To the best of our knowledge, this is the first example of the integration of multiple biomimetic catalysts through a controlled spatial positioning procedure. Meanwhile, our new findings might pave the way to applying artificial tandem catalytic systems for artificially mimicking organelles or important chemical transformations.

## Results and discussion

### Synthesis of GSHA

**Fig. 2:**
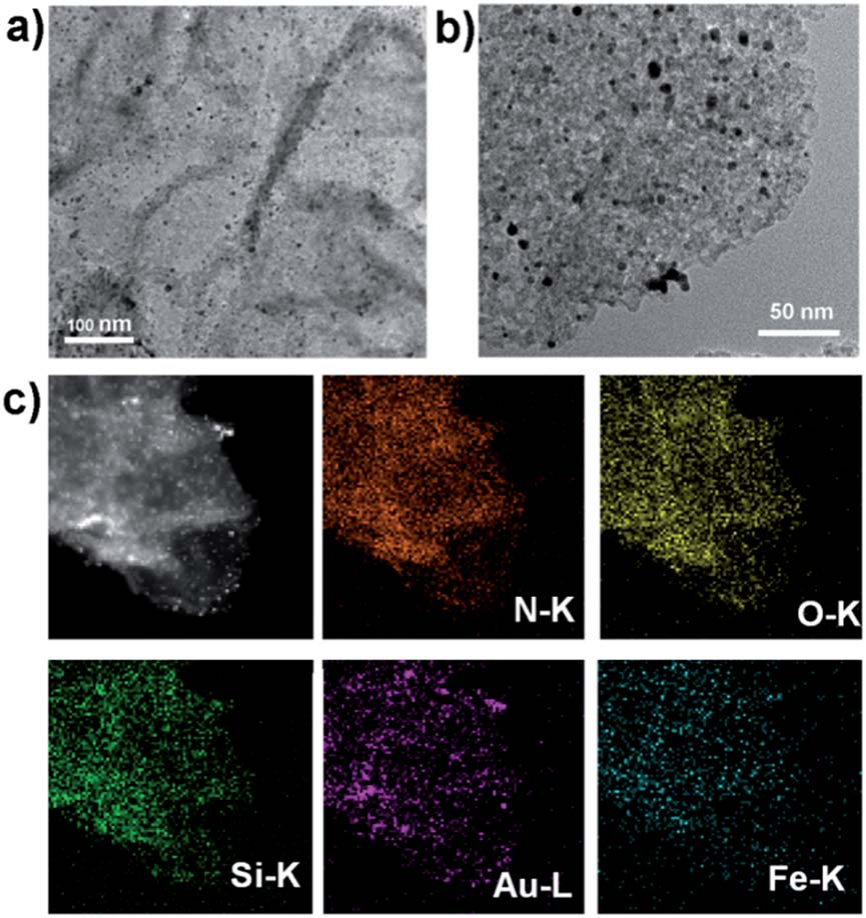
(a and b) TEM images of GSHA sheets. (c) Dark-field TEM image, and corresponding TEM elemental mappings of the N K-edge, O K-edge, Si K-edge, Au L-edge and Fe K-edge signals.

Fig. 1A illustrates the basic procedure using the GS as a scaffold for the precise positional assembly of hemin and AuNPs to form GS–hemin–AuNPs nanohybrids (GSHA). The structure of hemin and the corresponding TEM images are shown in Fig. 1B and C. Firstly, the 2D sandwich-like GS was prepared by Feng and Müllen's recently developed method with a slight modification.^18^ Briefly, the cationic surfactant, cetyltrimethylammonium bromide (CTAB), electrostatically adsorbed and self-assembled onto the surface of highly negatively charged graphene oxide (GO) in alkaline solution. Upon the hydrolysis of tetraethyl orthosilicate (TEOS), hydrazine reduction treatment, surface functionalization with amine groups (*i.e.* APTES treatment) and soft-template removal, the GS products were successfully collected with mesoporous silica around the surface of single-layer graphene. The resulting GS products were studied by transmission electron microscopy (TEM) imaging. As seen in Fig. 1C and S1,† as-prepared GS sheets with a morphology similar to that of graphene and a mesoporous structure were observed.^18^ Additionally, atomic force microscopy (AFM) was conducted to further demonstrate the structural features of the GS sheets (Fig. S2†). Then, as a flat molecule, hemin (a well-known natural metalloporphyrin) could be assembled onto the surface of the exposed graphene to form GS–hemin (GSH) through π–π stacking interactions.^15^ The conjugation experiment between hemin and the GS was carried out in methanol solution, as hemin is monomeric under this condition (Fig. S3a†). Although it is difficult to distinguish between the GS and GSH using TEM (Fig. 1C), the attachment of hemin on the exposed graphene surface could be characterized by UV/vis absorption spectroscopy. As shown in Fig. S3b,† an absorption maximum at 418 nm was also observed after reduction, which clarified that the hemin molecules were attached to the exposed graphene.^15a,b^ The next step was to adsorb AuCl_4_^–^ to the NH_2_-group-rich silica surface of GSH *via* electronic interactions. After that, highly dispersed AuNPs could be formed on the silica surface (GSHA) by *in situ* reduction of auric chloride ions with NaBH_4_, since the functional NH_2_ groups present could serve as a stabilizing agent by providing an anchoring surface.^19^ The nature of the GSHA structure was studied by TEM and high-angle annular dark-field scanning TEM (HAADF-STEM) and elemental mapping (Fig. 1C and 2). Uniform distribution of N, O, Si, Au and Fe in the same graphene support was observed, which indicated that the AuNPs and hemin had been co-immobilized into the same GS support. Such a hybrid catalyst based on heterogeneous materials contained different catalytic species, which were expected to possess multiple enzyme-like activities. Similarly, GS–AuNPs nanohybrids (GSA) and GS–AuNPs–hemin nanohybrids (GSAH) were also prepared (Scheme S1, S2†). Characterizations of the resulting GSA and GSAH are described in detail in the ESI (Fig. S4 and S5†). Compared to anchoring the AuNPs prior to the introduction of hemin (GSAH), the first way, creating a catalytic GSHA ensemble, was thought to be more practical, as it was demonstrated that hemin could also adsorb onto the gold surface during the hemin assembly process (Fig. S6†).

### The peroxidase-like catalytic activity of GSH

**Fig. 3:**
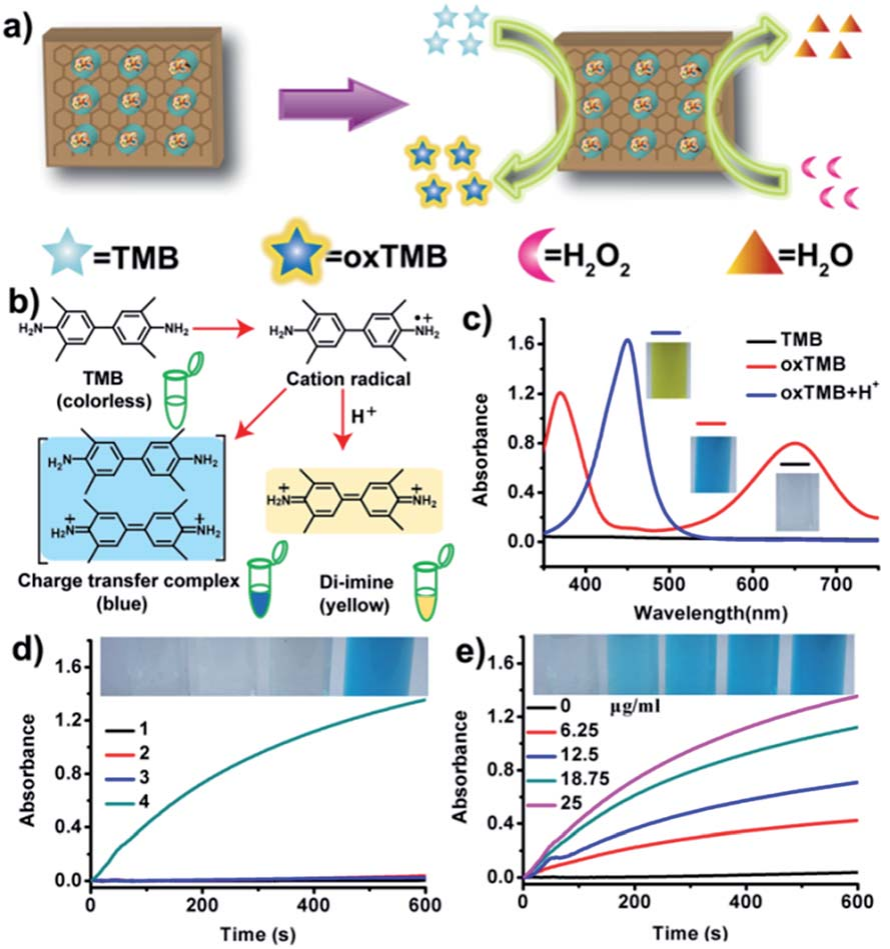
The peroxidase-like catalytic activity of GSH sheets. (a) Schematic illustration of GSH-catalyzed TMB oxidation to produce oxTMB. (b) TMB oxidation pathways and their corresponding chemical structures. (c) UV-vis absorbance spectra of TMB and its oxidation products. (d) Time-dependent absorbance changes at 652 nm for different samples after incubation with TMB: (1) none; (2) only H_2_O_2_; (3) GSH alone; (4) H_2_O_2_ and GSH. Inset: corresponding visual color changes ([TMB] = 1 mM, [H_2_O_2_] = 50 mM, [GSH] = 25 μg mL^–1^). (e) Time-dependent absorbance changes in the absence or presence of different concentrations of GSH. Inset: corresponding visual color changes.

To demonstrate the proof of principle, the peroxidase-like catalytic activity of GSH was first investigated under different conditions (Fig. 3), compared with that of hemin alone or GSA. Because of molecular aggregation and oxidative destruction, free hemin itself is generally inactive as a catalyst. After the assembly process, the adsorbed hemin species on the exposed graphene surface of the GS is monomeric and can function as a highly effective catalyst in various biomimetic oxidation reactions. For instance, like horseradish peroxidase (HRP) (Fig. S7†), it can catalyze the reaction of the peroxidase substrate 3,3,5,5-tetramethylbenzidine (TMB) in the presence of H_2_O_2_ (Fig. 3a). The TMB oxidation pathways by GSH/H_2_O_2_ can be described as shown in Fig. 3b. As expected, GSH had high catalytic activity, whereas free hemin showed little activity at the same hemin concentration (Fig. S8†). The oxidation of TMB produced a blue color with major absorbance peaks at 370 and 652 nm (Fig. 3c). After incubation of sulfuric acid, the reaction was stopped, and the blue color changed to yellow with maximum absorbance at 450 nm (Fig. 3c). Control studies indicated that neither H_2_O_2_ nor GSH alone could efficiently oxidize TMB (Fig. 3d). Meanwhile, the ability of GSH/H_2_O_2_ to oxidize TMB was dependent on catalyst concentration (Fig. 3e) and pH (Fig. S9†).

### The glucose oxidase-mimic activity of GSA

**Fig. 4:**
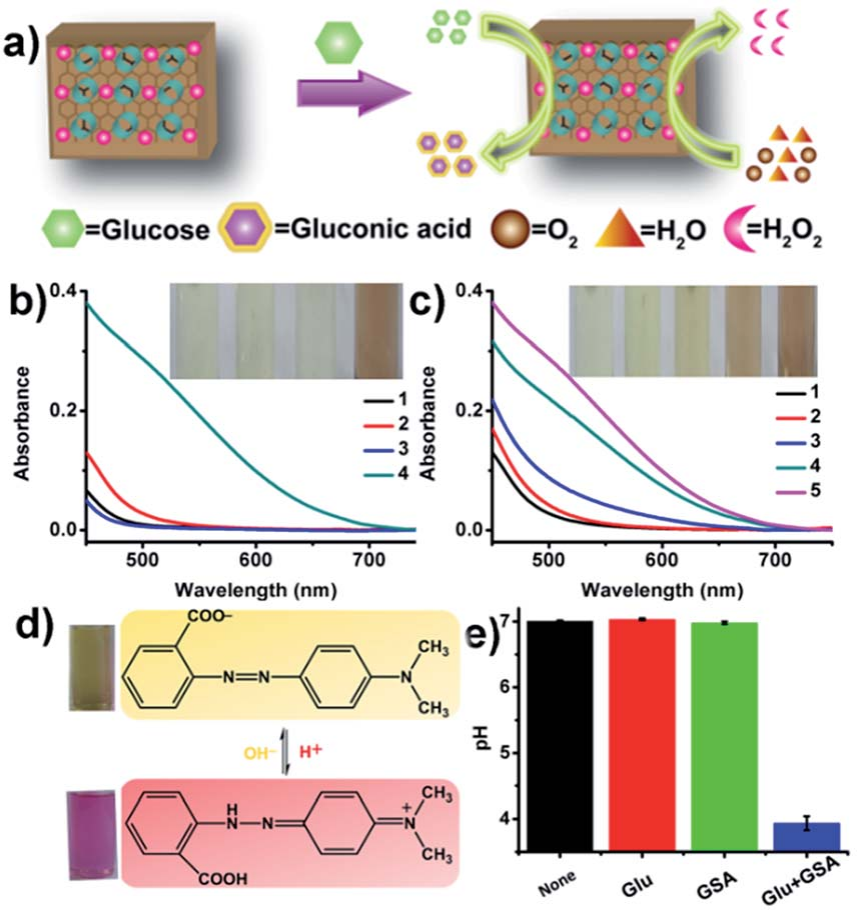
The GO*x*-like catalytic activity of the GSA sheets. (a) Schematic illustration of GSA-catalyzed glucose oxidation to produce gluconic acid and H_2_O_2_. (b) Relative absorbance spectra and visual color changes for different samples obtained by a gluconic acid-specific assay: (1) none; (2) only glucose; (3) GSA alone; (4) glucose and GSA ([glucose] = 200 mM, [GSA] = 900 μg mL^–1^). (c) Relative absorbance spectra and visual color changes in the absence or presence of different concentrations of GSA (1 to 5: 0, 100, 300, 600, 900 μg mL^–1^). (d) Typical photographs and corresponding chemical structures of methyl red without or with glucose and GSA in phosphate buffer (0.5 mM, pH 7.0). (e) pH change for different samples in phosphate buffer (0.5 mM, pH 7.0).

Next, we systematically evaluated the glucose oxidase-mimic activity of GSA in solution (Fig. 4). As unsupported AuNPs have recently been found to exhibit intrinsic GO*x*-like activity,^11a–d^ we reasoned that the “naked” AuNPs supported on the GS could serve as a more effective GO*x* mimic. Like GO*x* (Fig. S10†), GSA could catalyze the oxidation of glucose by means of molecular oxygen (in equilibrium with air), yielding gluconic acid and H_2_O_2_ (Fig. 4a). The reaction solution was studied with a gluconic acid-specific colorimetric assay.^11*a*^ Upon the addition of hydroxamine and Fe^3+^, the color of the solution turned red with a characteristic absorbance peak at 505 nm (Fig. 4b and c), which suggested that gluconic acid was indeed produced in this GSA-catalyzed reaction. The solutions containing glucose or GSA alone could not introduce any color change. However, control experiments indicated that GSH without AuNPs and citrate-capped AuNPs (13 nm) had very little activity. This is because the GS support helps the formation of a high degree of ultrafine AuNPs (Fig. S4†). As a result, a larger fraction of active metal atoms are exposed to the surface, and thus these very small and stable AuNPs possess highly enhanced catalytic activity.^11*a*^ In addition, since gluconic acid is one of the organic acids, we reason that its production in the reaction can also decrease the ambient pH. To further confirm the reaction product, we used methyl red as a pH indicator (red in pH under 4.4 and yellow in pH over 6.2) and a pH meter to monitor the pH change of the solution (Fig. 4d and e). All the above results confirmed that GSA can act as a more effective GO*x* mimic than unsupported AuNPs.^11a–d^

### GSHA-catalyzed two-step reaction

**Fig. 5:**
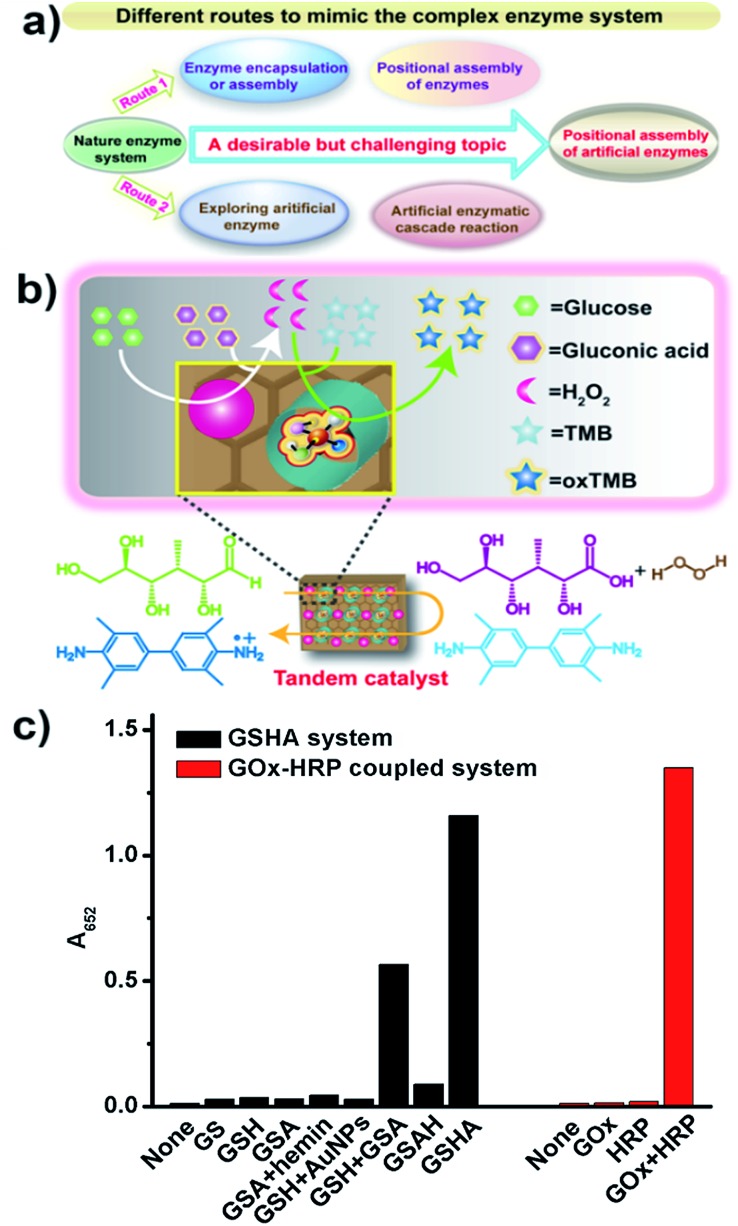
(a) Current status and future challenges for mimicking the complex enzyme system. (b) Schematic illustration of a two-step reaction taking place in the GSHA system. (c) Formation of oxTMB in the GSHA or oxidase–peroxidase coupled enzyme system in phosphate buffer (0.5 mM, pH 7.0), ([TMB] = 1 mM, [GS, GSA, GSH, GSAH, GSHA] = 1 mg mL^–1^, [glucose] = 200 mM for artificial enzyme or 4 mM for natural enzyme, [GO*x*] = 100 μg mL^–1^, [HRP] = 1 ng mL^–1^).

So far, many studies have been reported in the literature with the objective of mimicking natural enzyme architectures. In terms of these studies, there are basically two major aspects concerning the construction of synthetic systems, namely (1) using enzyme encapsulation or assembly to mimic the natural compartmentalisation process (Fig. 5a, Route 1);^1–3,5^ such a strategy has been developed to encapsulate and position different types of natural enzymes in separate domains, and (2) exploring artificial enzymes that mimic the function of natural enzymes (Fig. 5a, Route 2);^8–15^ researchers have recently directed their attention to the construction of catalytic ensembles for mimicking complex enzymatic systems.^15b,17^ However, there has been no report of artificial enzyme-loaded nanodevices with a high level of control over positional assembly for mimetic tandem catalysis. Based on the enzyme-mimicking activities of GSH and GSA, we expected that the integrated GSHA could function as a hybrid catalyst that could drive a two-step reaction to allow for *in situ* generation of H_2_O_2_ for the oxidation of the peroxidase substrate TMB (Fig. 5b). Initially, the enzyme-like activities of GSHA were tested separately. GSHA could catalyze the oxidation reaction of both glucose and TMB (Fig. S12†). In contrast, GSA or GSH could only possess one of the enzyme-like activities under our experimental conditions. These above experimental results demonstrated that GSHA could exhibit dual enzyme-mimicking activities. Inspired by these unique features, we further pieced them together to catalyze a two-step reaction, usually catalyzed by GO*x* and HRP. That is, GSHA first catalyzed the glucose oxidation reaction to yield gluconic acid and H_2_O_2_, and then oxidized TMB resulting in the formation of a colored product of oxTMB [eqn (1)]:1
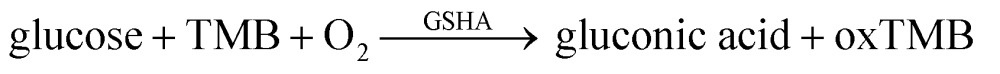



As shown in Fig. 5c and S13,† similar with the oxidase–peroxidase coupled enzyme system, GSHA could produce a blue color due to the oxidation of TMB by H_2_O_2_, indicating that the entire reaction was operational in solution. Control experiments confirmed that neither GSA nor GSH could catalyze the TMB oxidation reaction (Fig. 5c and S13†), unless their catalytic reactions were coupled with enzymatic catalysis (Fig. S14 and S15†). More importantly, positional assembly of hemin and AuNPs in spatially separate domains (GSHA) shows a clear advantage over other similar systems without such an assembly (Fig. 5c and S13†). For example, GSAH almost cannot catalyze this two-step cascade reaction (Fig. 5c and S13†), as the hemin adsorbed onto the gold surface can inhibit the GO*x*-like activity of GSAH. Taken together, artificial enzyme complexes with significantly improved compositional and spatial controls were developed for realizing more complex functions.

## Conclusion

In conclusion, we have demonstrated that it is possible to find a suitable support and to entrap hemin molecules and AuNPs within the support in a controlled way. Addition of glucose to a dispersion of such a multifunctional hybrid catalyst resulted in tandem catalysis, which was commonly completed by the oxidase–peroxidase coupled enzyme system. Overall, our studies show a general strategy to position artificial enzymes with different functions into a unique support, which holds great promise for designing other hybrid catalysts with a high level of control. This will be important for the future development of catalytic ensembles that can function as “artificial organelles” or enable important chemical transformations not otherwise readily possible.
