# Supplementary material for: Positional assembly of hemin and gold nanoparticles in graphene–mesoporous silica nanohybrids for tandem catalysis
Source: Chem Sci. 2014 Nov 24;6(2):1272–6. doi: 10.1039/c4sc02714k (PMC5811075; doi:10.1039/c4sc02714k)
Supplement: Supplementary file 1 [file SC-006-C4SC02714K-s001.pdf]

Electronic Supplementary Material (ESI) for Chemical Science.
This journal is © The Royal Society of Chemistry 2014

Supporting Information

Positional Assembly of Hemin and Gold Nanoparticles in Graphene-Mesoporous Silica Nanohybrids for Tandem Catalysis

Youhui Lin, Li Wu, Yanyan Huang, Jinsong Ren,* and Xiaogang Qu

Experimental Section

Reagents and materials: Graphite, glucose and hydrogen tetrachloroaurate (III) ($HAuCl_4 \cdot 3H_2O$) were purchased from Sinopharm ChemicalReagent (Shanghai, China). Tetraethylorthosilicate (TEOS), sodium hydroxide and 1,3,5-trimethylbenzene were purchased from Sigma-Aldrich. 3,3,5,5-Tetramethylbenzidine (TMB) was purchased from BBI (Ontario, Canada). Hydroxylamine, N-cetyltrimethylammonium bromide (CTAB), 3-aminopropyltriethoxysilane (APTES), and sodium borohydride ($NaBH_4$) were obtained from Alfa Aesar. Hydrazine (85%), hydrochloric acid, and H_2O_2 were obtained from Beijing Chemicals (Beijing, China). Horseradish peroxidase and glucose oxidase was obtained from by Shanghai Sangon Biological Engineering Technology & Services (Shanghai, China). All other reagents were of analytical reagent grade, and used as received. Ultrapure water (18.2 MΩ; Millpore Co., USA) was used throughout the experiment.

Apparatus and Characterization: Atomic force microscopy (AFM) measurements were recorded using Nanoscope V multimode atomic force microscope (Veeco Instruments, USA). The UV-Vis absorption

spectra were performed using a JASCO V-550 UV/Visible spectrophotometer (JASCO International Co., LTD., Tokyo, Japan). SEM images were obtained with a Hitachi S-4800 FE-SEM. TEM images and high-angle annular dark-field scanning TEM (HAADF-STEM) were performed using a FEI TECNAI G2 20 high-resolution transmission electron microscope operating at 200 kV. The crystalline structures of the as prepared samples were evaluated by X-ray diffraction (XRD) analysis on a Rigaku-Dmax 2500 diffractometer by using CuKα radiation. The operation voltage and current were kept at 40 kV and 40 mA.

Synthesis of graphene-mesoporous silica hybrid (GS): The procedure for the synthesis of graphene oxide (GO) was followed by the modified Hummers method.[1] Then, GS was synthesized from GO by the literature with some modification.[2] Briefly, 5.8 mL the as-prepared GO (3.8 mg/mL) solution was added into 44.2 mL water containing 20 mg NaOH and 0.5 g CTAB. After ultrasonically treating for 1 h and magnetic stirring for 2 h at 40 °C, 2 mL TEOS solution (400 μL dissolved in 1.6 mL ethanol) was slowly introduced into the above mixture. After the hydrolysis of TEOS for 12 h, 80 μL hydrazine was additionally added to the above mixture, and then heated at 70 °C for another 5 h. The obtained product was centrifuged and washed with warm ethanol for three times. After that, the product was mixed with 200 μL APTES in 50 mL ethanol and stirred for12 h at 80 °C under reflux before centrifugation. The obtained product was then dispersed in50 mL acetone stirred at 40 °C for 24 h. Finally, the GS product was collected by centrifugation and washed by warm ethanol for three times.

*Synthesis of GS-supported catalysts(GSH, GSA, GSAH, GSHA):*Taking GSHA catalyst as an example, the resulting GS was re-dispersed in 0.5 mM or 20 μM hemin-containing methanol solution (20 μM hemin was used for artificial enzymatic cascade system as excess hemin will result in an excessive background of oxTMB; and 0.5 mM hemin were used for all of other experiments). Due to the

insolubility of hemin in neutral aqueous solution, our conjugation experiments between hemin and GS support were carried out in methanol solution. The mixture was stirred mildly for 2 hours to allow the interaction of hemin and GS. After that, GSA was collected by centrifugation and washed by methanol for several times.

The obtained GSH (10 mg) were dispersed in 1 mL distilled water by sonication for 20 min. Next, the $HAuCl_4$ solution (0.1 mL; 20 mM) was added into the above aqueous solution. After 10 min sonication, a freshly prepared $NaBH_4$ (0.5 mL; 0.1 M) was added into the above mixture under vigorous stirring. After mixing, the resulting solution was stirred for another 30 min. Finally, the GSHA product was collected by centrifugation and washed by water. Similarly, GSH, GSA and GSAH can be synthesized.

Peroxidase-like activity: Activity assay of different catalysts was performed by the published test procedure.[3] The oxidation of TMB can result in a blue color with major absorbance peaks at 370 and 652 nm. Our experiments were carried out using different catalysts in a reaction volume of 400 μL 25 mM phosphate buffer with 1 mM TMB, 50 mM H_2O_2, unless otherwise stated. After mixing, monitoring the formation of the radical cation of TMB by its absorption at 652 nm was started immediately.

Glucose-oxidase-like activity: Different catalysts and glucose (200 mM) in 5 mM phosphate buffer (pH 7.0) were incubated at 35 °C for 8h, and their catalytic activities are assayed by a gluconic acid-specific colorimetric assay.[4] The interaction of gluconic acid, hydroxylamine and Fe^{3+} leads to a red complex with a major absorbance at 505 nm. Briefly, the 400 μL of above solutions were added into 250 μL of solution 1 (0.15 mM triethylamine in water and 5 mM EDTA) and 25 μL of solution 2 (3 M NH_2OH in water). After 25 min of incubation, 125 μL of solution 3 (0.1 M $FeCl_3$, 1 M HCl, and 0.25 M

CCl_3COOH in water) was introduced into the above mixture. After mixing, the reaction was allowed to proceed for another 5 min. Before spectral measurements, different GS-supported catalysts were centrifuged in experiments, to prevent the influence of the absorbance of GS-supported catalysts to the colorimetric reaction.

An artificial enzymatic cascade system: Briefly, 1 mg/mL GS-supported catalysts (or unsupported AuNPs or hemin molecules) were mixed with 200 mM glucose in 0.5 mM phosphate buffer (pH 7.4) at 35 °C, and 40 mM TMB (10 μL) was added into the reaction solution (400 μL).The product of this cascade reaction (oxTMB) was assayed by its major absorbance peaks at 652 nm.

[1] W. S. Hummers , R. E. Offeman , *J. Am. Chem. Soc. 1958*, **80**, 1339.
[2] S. Yang, X. Feng, L. Wang, K. Tang, J. Maier, K. Müllen, *Angew. Chem. 2010*, **122**, 4905–4909; *Angew. Chem. Int. Ed.* 2010, **49**, 4795-4799.
[3] Y. Liu, J. Du, M. Yan, M. Y. Lau, J. Hu, H. Han, O. O. Yang, S. Liang, W. Wei, H. Wang, J. Li, X. Zhu, L. Shi, W. Chen, C. Ji, Y. Lu, *Nat. Nanotechnol. 2013*, **8**, 187-192.
[4] W. Luo, C. Zhu, S. Su, D. Li, Y. He, Q. Huang, C. Fan, *ACS Nano 2010*, **4**, 7451-7458.

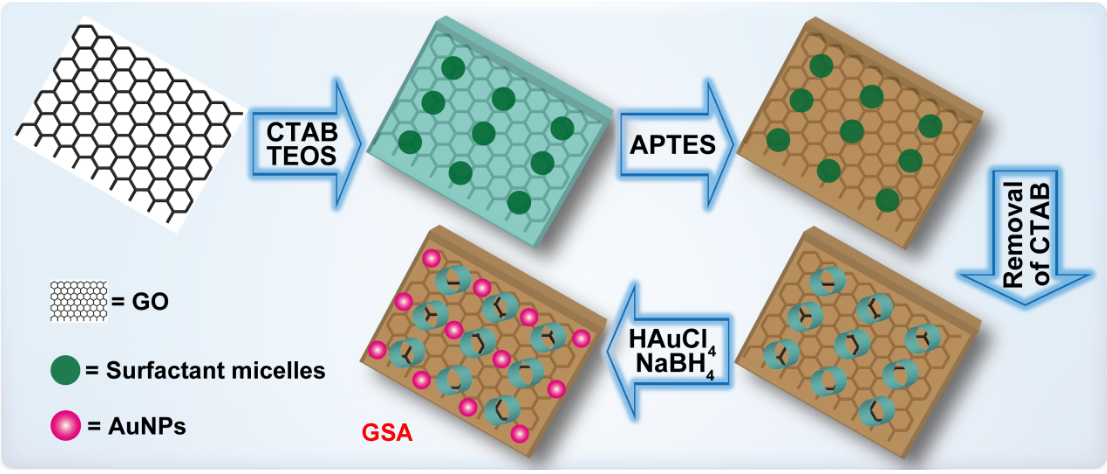

Scheme S1. Synthetic strategy for the construction of GSA catalyst.

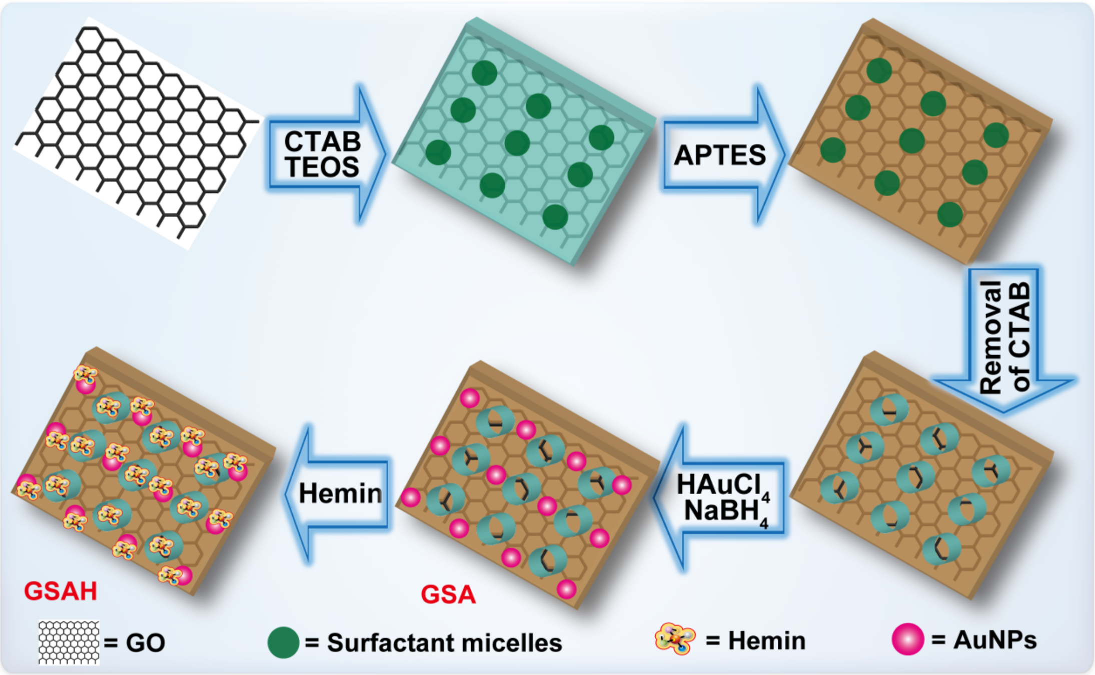

Scheme S2. Synthetic strategy for the construction of GSAH catalyst.

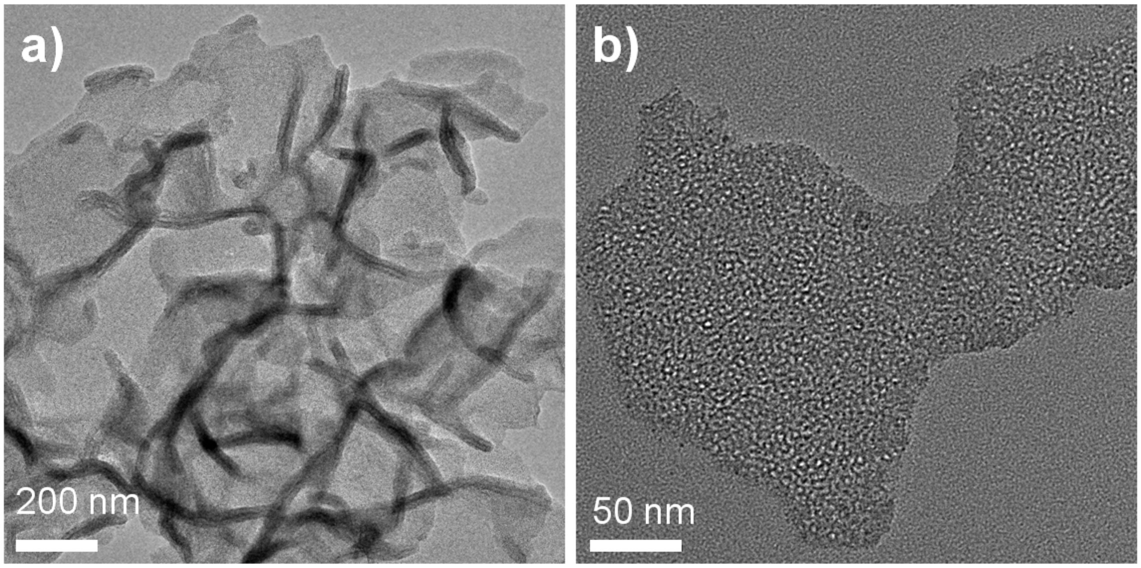

Fig. S1 Low magnification (a) and high-resolution (b) TEM image of GS.

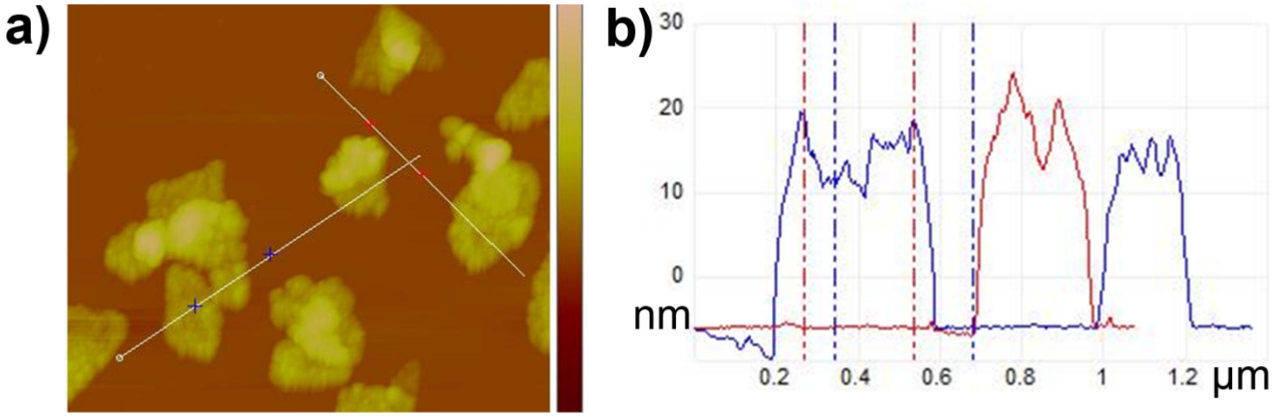

Fig. S2 (a) AFM image and (b) height profile of GS deposited on mica substrates. (AFM image and thickness analyses reveal the same morphology observed by TEM)

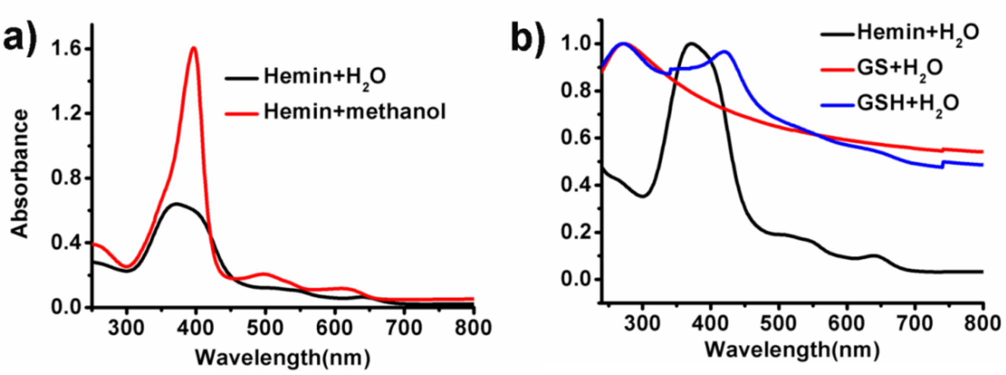

Fig. S3 (a) UV/Vis spectroscopy of free hemin in H_2O (black line) and methanol solution (red line). (b) UV/Vis spectroscopy of free hemin in H_2O (black line), GS in H_2O (red line), and separated GSH redispersed in H_2O solution (blue line).

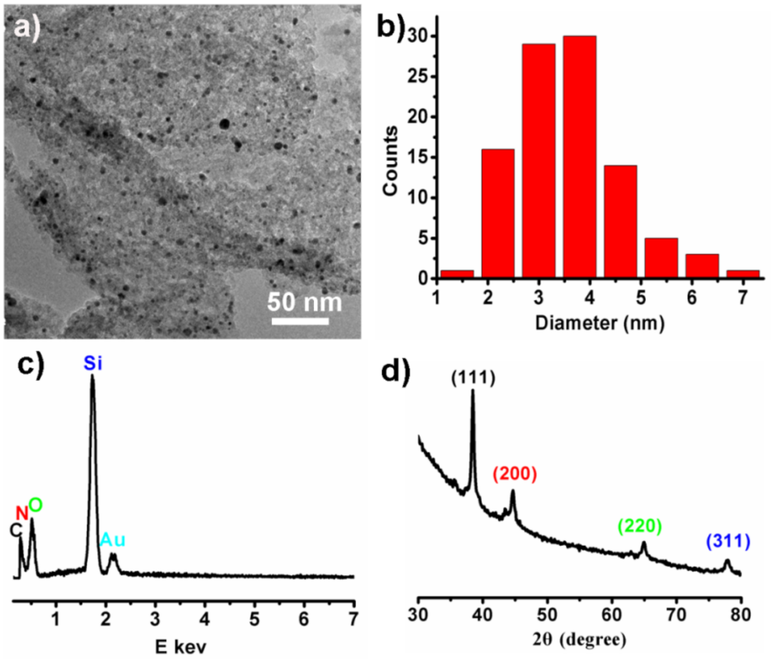

Fig. S4 (a) TEM image of GSA. (b) Size distribution histogram of AuNPs in GSA. The total number of AuNPs counted for the histogram was 100. (c) The energy-dispersive X-ray (EDX) analysis spectrum of GSA. (d)Wide-angle powder XRD analysis spectrum of GSA. (Four peaks of GSA in XRD analysis spectrum are consistent with the (111), (200), (220), and (311) reflections of the face-centered-cubic structure of crystalline Au^0).

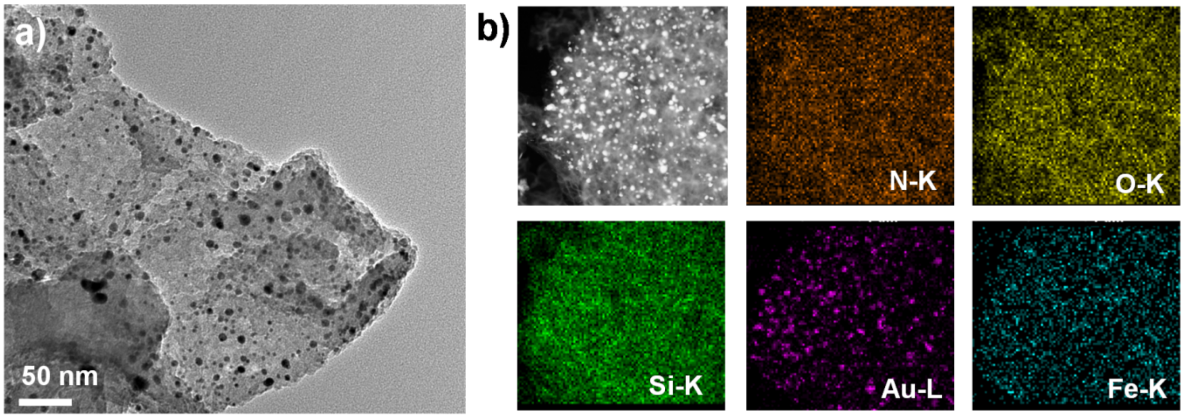

Fig. S5 (a) TEM image of GSAH sheets. (b) Dark-field TEM image, and corresponding TEM elemental mappings of the N K-edge, O K-edge, Si K-edge, Au L-edge and Fe K-edge signals.

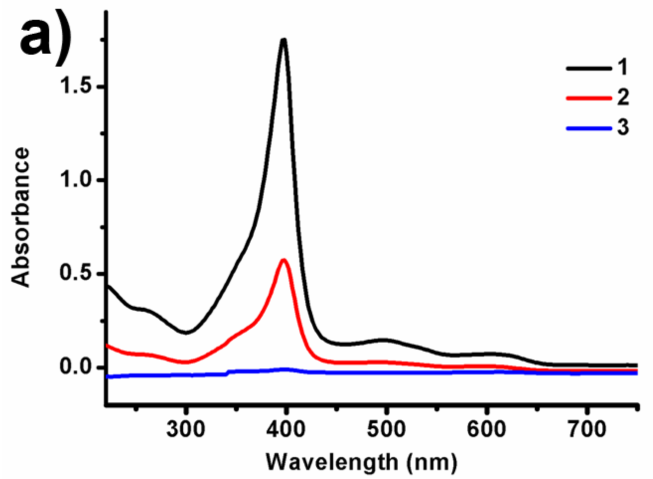

b)

	Atom. [at. %]
Fe (GSH)	0.27%
Fe (GSAH)	1.0%

Fig. S6 (a) The absorbance of hemin in different samples: 1) 10 μL hemin (0.5 mM) in 400 μL methanol; 2) 10 μL supernatant of hemin-GS conjugation system in 400 μL methanol; 3) 10 μL supernatant of hemin-GSA conjugation system in 400 μL methanol. (The amount of hemin left in the supernatant indicated more hemin adsorb onto the GSA than GS.) (b) Fe element analysis of the GSH and GSAH samples by EDX analysis. Both experiments indicated that hemin could adsorb onto gold surface.

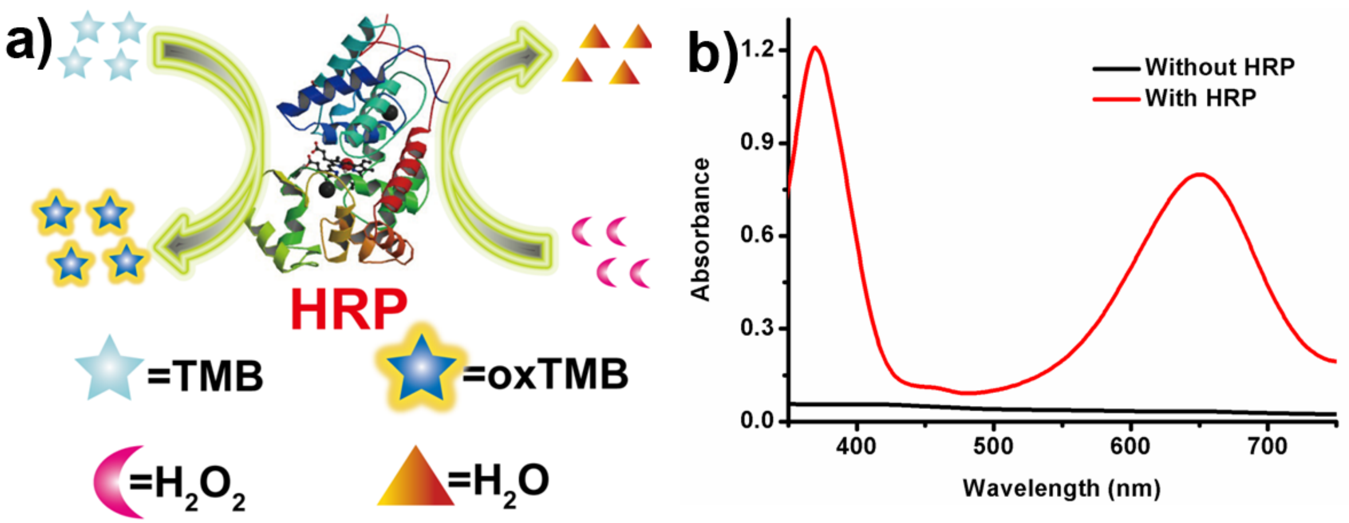

Fig. S7 The catalytic activity of natural enzyme HRP. (a) Schematic illustration of HRP catalyzed TMB oxidation to produce oxTMB. (b) The absorbance change as a result of the catalyzed oxidation of TMB in the presence of H_2O_2.

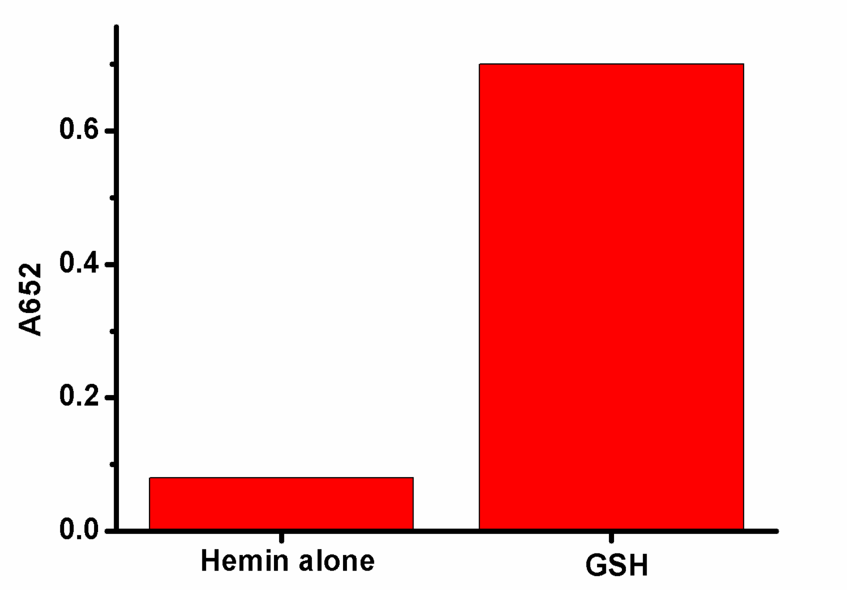

Fig. S8 The absorbance change as a result of the catalyzed oxidation of TMB (the same hemin concentration of hemin and GSH was used in experiments).

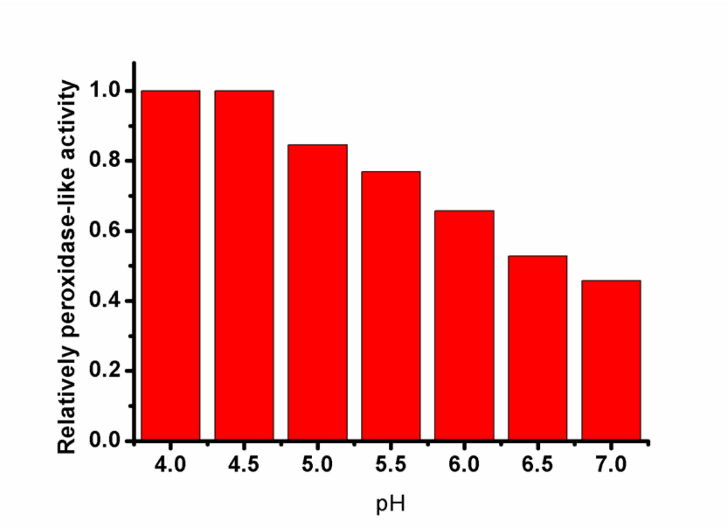

Fig. S9 pH-dependent peroxidase-like activity of GSH.

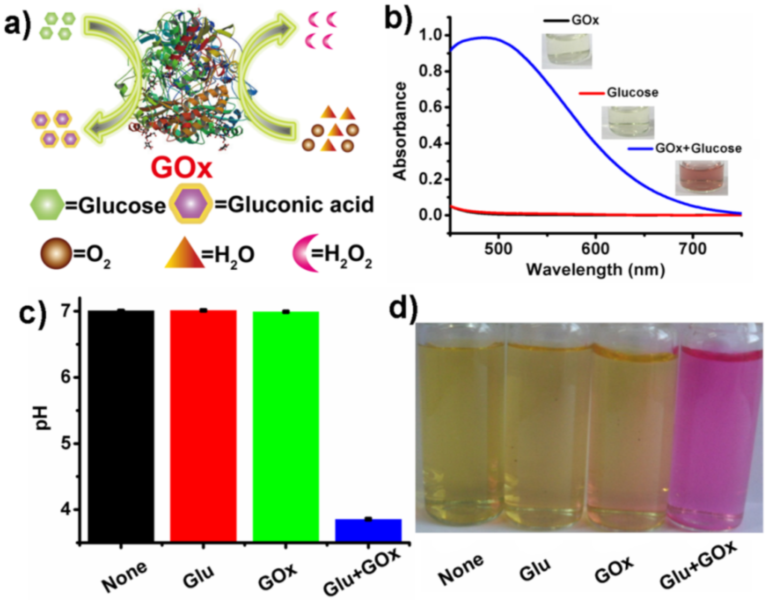

Fig. S10 The catalytic activity of GOx. (a) Schematic illustration of GOx-catalyzed glucose oxidation to produce gluconic acid and hydrogen peroxide. (b) Relative absorbance spectra and visual color changes for different samples obtained by gluconic acid-specific assay: GOx alone (black line); only glucose (red line); glucose and GOx (blue line). ([glucose] =5 mM, [GOx] =200 μg/mL). (c) pH changes for different samples in phosphate buffer (0.5 mM, pH 7.0). (d) Typical photographs of methyl red for different samples in phosphate buffer (0.5 mM, pH 7.0).

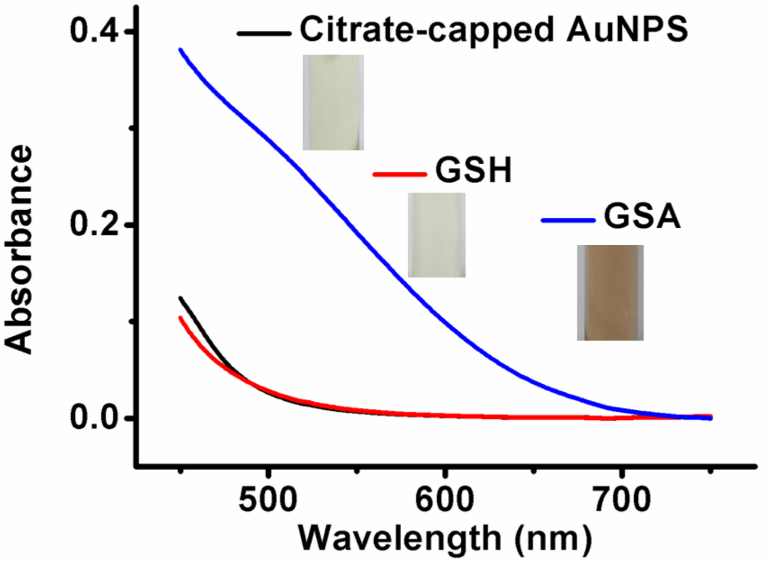

Fig. S11 Relative absorbance spectra and visual color changes for different samples obtained by gluconic acid-specific assay: 1) glucose and citrate-capped AuNPs; 2) glucose and GS; 3) glucose and GSA. ([glucose] =200 mM, [GSA, GS] = 900μg/mL). (The amount of Au atoms in citrate-capped AuNPs (13 nm) or GSA is the same.)

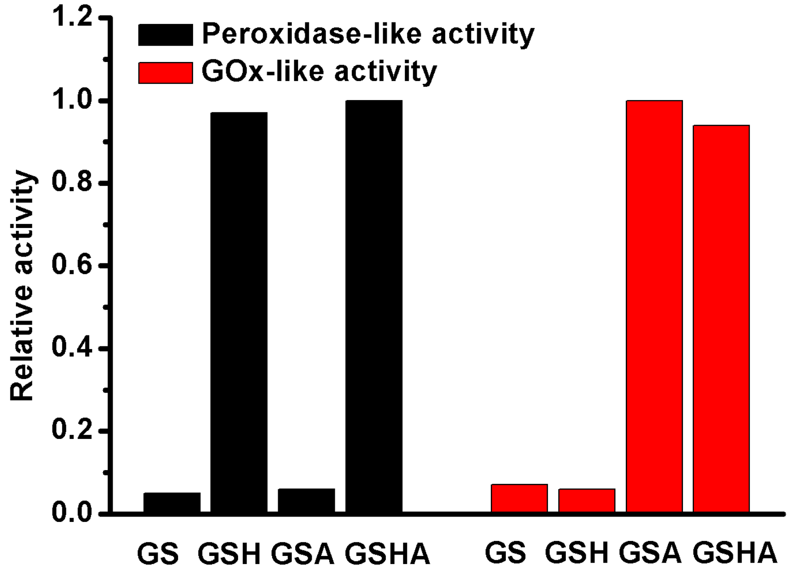

Fig. S12 Comparison of the peroxidase-(a) or GOx-like (b) activity of different GS-support catalysts.

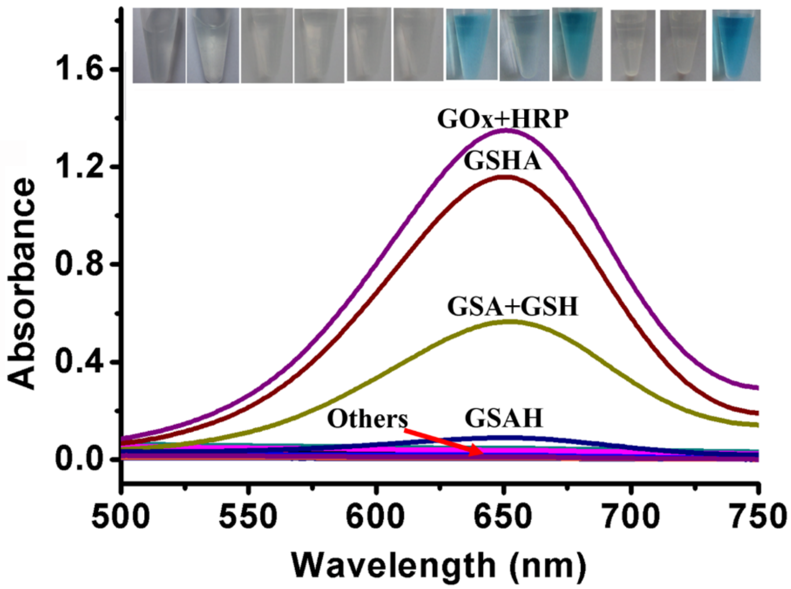

Fig. S13 The UV/Vis absorption spectra and corresponding color changes as a result of the formation of oxTMB by different catalysts: 1) None; 2) GS; 3) GSH; 4) GSA; 5) GSA + hemin; 6) GSH + unsupported AuNPs; 7) GSH +GSA; 8) GSAH; 9) GSHA; 10) GOx; 11) HRP; 12) GOx + HRP. (Before measurements, all the samples were centrifuged to prevent the influence of the absorbance of prepared catalysts to the colorimetric reaction.)

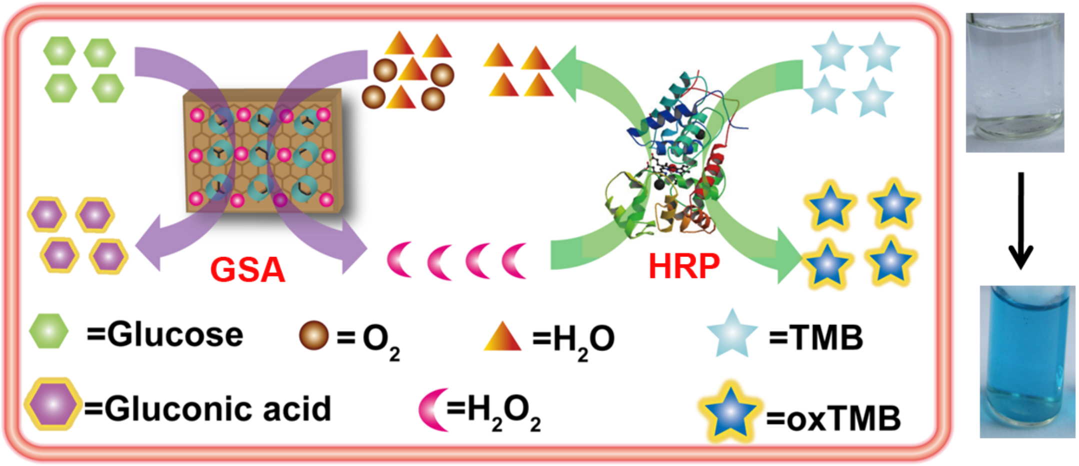

Fig. S14 GSA catalyze the reduction of O_2 to H_2O_2, followed by a HRP-based catalytic reaction, resulting in a blue colored product of oxTMB.

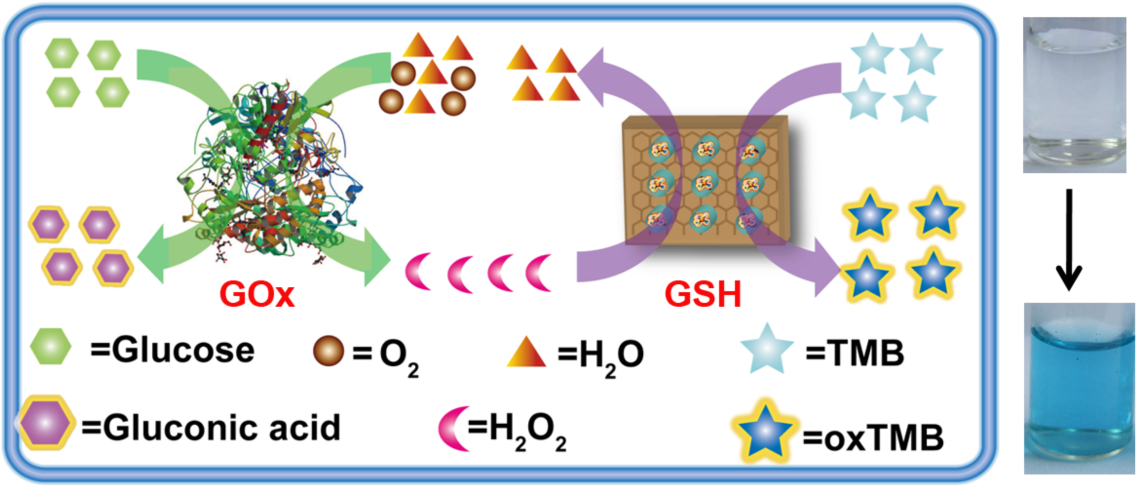

Fig. S15 GOx catalyze the reduction of O_2 to H_2O_2, followed by a GSH-based catalytic reaction, resulting in a blue colored product of oxTMB.
